# Impact of deformable image registration on dose accumulation applied electrocardiograph-gated 4DCT in the heart and left ventricular myocardium during esophageal cancer radiotherapy

**DOI:** 10.1186/s13014-018-1093-z

**Published:** 2018-08-10

**Authors:** Ying Tong, Yong Yin, Pinjing Cheng, Guanzhong Gong

**Affiliations:** 1 Radiation Physics Department of Shandong Cancer Hospital Affiliated to Shandong University, Jinan, China; 2 School of Nuclear Science and Technology, University of South China, Hengyang, China

**Keywords:** Esophageal cancer radiotherapy, Deformable image registration, Dose-volume parameters, Heart, Left ventricular myocardium

## Abstract

**Background:**

The deformable image registration (DIR) technique has the potential to realize the dose accumulation during radiotherapy. This study will analyze the feasibility of evaluating dose-volume parameters for the heart and left ventricular myocardium (LVM) by applying DIR.

**Methods:**

The electrocardiograph-gated four-dimensional CT (ECG-gated 4DCT) data of 21 patients were analyzed retrospectively. The heart and LVM were contoured on 20 phases of 4DCT (0%, 5%,…,95%). The heart and LVM in the minimum volume/dice similarity coefficient (DSC) phase (Volume _min_/DSC _min_) were deformed to the maximum volume/DSC phase (Volume _max_/ DSC _max_), which used the intensity-based free-form DIR algorithm of MIM software. The dose was deformed according to the deformation vector. The variations in volume, mean dose (D_mean_), V_20_, V_30_ and V_40_ for the heart and LVM before and after DIR were compared, and the reference phase was the Volume _max_/DSC _max_ phase.

**Results:**

For the heart, the difference between the pre- and post-registration Volume _min_ and Volume _max_ were reduced from 13.87 to 1.72%; the DSC was increased from 0.899 to 0.950 between the pre- and post-registration DSC _min_ phase relative to the DSC _max_ phase. The post-registration D_mean_, V_20_, V_30_ and V_40_ of the heart were statistically significant compared to those in the Volume _max_/DSC _max_ phase (*p* < 0.05). For the LVM, the difference between the pre- and post-registration Volume _min_ and Volume _max_ were only reduced from 18.77 to 17.38%; the DSC reached only 0.733 in the post-registration DSC _min_ phase relative to the DSC _max_ phase. The pre- and post-registration volume, D_mean_, V_20_, V_30_ and V_40_ of the LVM were all statistically significant compared to those in the Volume _max_/DSC _max_ phase (*p* < 0.05).

**Conclusions:**

There was no significant relationship between the variation in dose-volume parameters and the variation in the volume and morphology for the heart; however, the inconsistency of the variation in the volume and morphology for the LVM was a major factor that led to uncertainty in the dose-volume evaluation. In addition, the individualized local deformation registration technology should be applied in dose accumulation for the heart and LVM.

## Background

Radiotherapy plays an important role in the treatment of thoracic tumors [1–3]. However, radiation-induced heart disease (RIHD) is a complication of radiotherapy [4–7]. Accurate evaluation of the cardiac dose can prevent the occurrence of RIHD. Kataria et al. found that cardiac activity led to a difference between the cardiac evaluation dose and cardiac actual dose, resulting in insufficient protection of the heart [8]. However, the use of electrocardiograph-gated four-dimensional CT (ECG-gated 4DCT) can provide a possibility for accurate calculation of the cardiac dose [9].

ECG-gated 4DCT combines the volume scan with the cardiac electrophysiological information, and the multi-sequence dynamic CT images showing the cardiac movements can be obtained by segmentation, which can capture the cardiac movements during the cardiac cycle [10–12]. In theory, the more phases of 4DCT images that are available can increase the sensitivity of the detection of cardiac activity and the accuracy of evaluation of the cardiac dose obtained by contouring the organs at risk (OAR) in all phases of 4DCT images and accumulating the doses in each phase. This approach may calculate the evaluation dose close to the actual dose; however, one previous study reported that this could be time-consuming [13].

The technique of deformable image registration (DIR) could be used to solve the above problems and realize the actual dose calculation of the heart. The DIR technique achieves point-to-point fusion of the target images and source images by looking for a space transform method [14]. Balik et al. indicated that the effect of DIR was better than that of rigid registration, and the dice similarity coefficient (DSC) could be up to 18.2% in comparison with that of the rigid registration [15].

However, the precondition of the actual dose accumulation calculation of the heart using the DIR technique is that the effect of the deformation is ideal. Therefore, in this study, the variation of dosimetry parameters (such as D_mean_, V_20_, V_30_ and V_40_) for the heart and the left ventricular myocardium (LVM), before and after deformation, was analyzed to explore the effect of deformation.

## Methods

### Patient selection

The ECG-gated 4DCT data of 21 patients based on breath-hold were analyzed retrospectively in this study, which were from March 2015 to November 2016. Of these patients, 11 patients were male, and 10 patients were female, with an age range of 35 to 67 years and a median age of 58 years old. All tumors that were evaluated in present study were esophageal tumors. In addition, this study was approved by the Research Ethics Board of the Shandong Cancer Hospital, and informed consent was obtained from all patients.

### Acquisition of 4DCT

All patients’ 4DCT images were acquired with a Siemens dual-source CT (Siemens SOMATOM Definition, DER). In addition, the images were then rebuilt via a 5% cardiac cycle; the 20 cardiac cycle images were rebuilt (0%, 5%, 10%⋯⋯95%) in this study, and all images were rebuilt at 0.75-mm slice thickness with an increment of 0.5 mm. The image resolution was 512 × 512, and the voxel size was 0.69 mm × 0.69 mm × 0.5 mm.

### Delineation of the heart and LVM

**Fig. 1:**
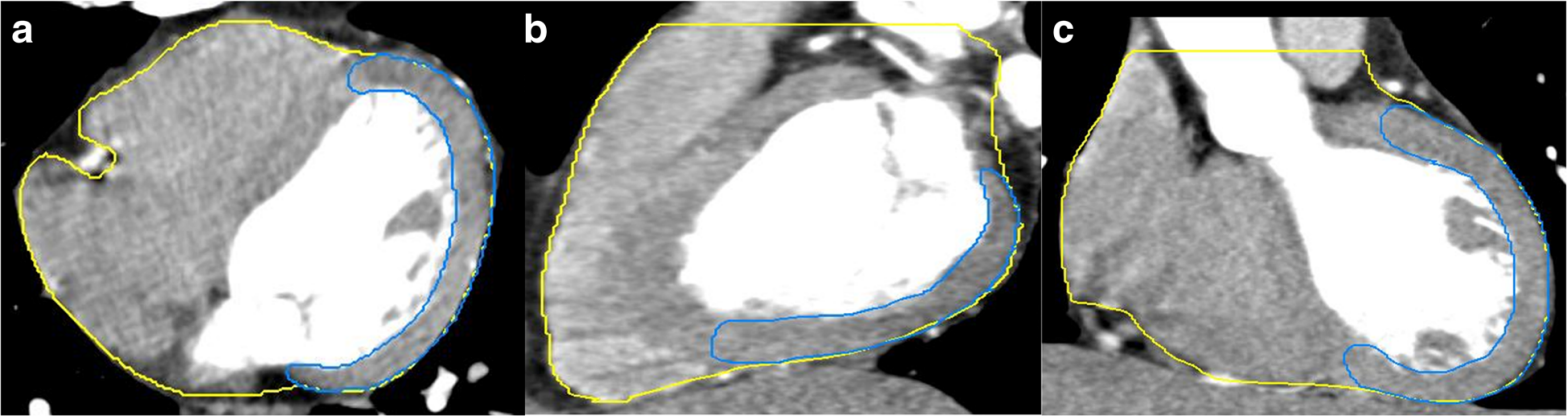
Delineation of the heart and left ventricular myocardium (LVM). **a** Delineation of the heart and LVM in transverse section. **b** Delineation of the heart and LVM in sagittal section. **c** Delineation of the heart and LVM in coronal section. Heart delineation is shown in yellow, and LVM delineation is shown in blue

The 4DCT images were imported in MIM Maestro 6.6.9 (MIM) (MIM Software Inc., America) workstation to contour the heart and LVM. In this study, the upper bound of the heart was the top of the left atrium, and the lower bound was the apex cordis; the upper bound of the LVM was the top of the left ventricle, and the lower bound was the apex cordis. The interventricular septum was not included, and the boundary between the LVM and interventricular septum was the left anterior descending coronary arteries. The window width/ window level was (400/40) HU (Fig. 1), and all delineations were performed by the same physician.

### Design plans

The radiotherapy plans were designed on the 0% phase images. The prescribed dose of the planning target volume (PTV) was 60 Gy for all plans. The dose distribution met the requirement that 95% of the PTV received the prescribed dose, and the constraints of the OAR were as follows: total lung V_20_ < 30%, V_30_ < 20%, maximum dose to the spinal cord < 45 Gy, the heart V_30_ < 40%, V_40_ < 30%.

### Deformable image registration

Most registration approaches can be classified as geometry-based or intensity-based. Geometry-based metrics make use of features extracted from the image data (anatomic/artificial landmarks or organ boundaries), while intensity-based metrics use the image voxel data directly [16]. All mathematical formulations of these similarity metrics are listed in Table 1 [16]. The commonly used transformation models mainly include the following [16]: (1) Rigid transformation: Allows for translation in 3 directions and rotations about 3 axes. (2) Affine transformation: Except for the translation/rotation, allows uniform scaling and sheer. (3) Free-form transformation: It is a local, voxel-based deformation, often regularized by a smoothing parameter, and this approach allows translation in 3 N dimensionality, where N is the number of voxels in an image. (4) Global Spline-based method transformation: Parameterizes deformation using a parametric grid of basis function control points with constrained global influence; the deformation is global, which allows translation in 3 N dimensionality. (5) Local spline-based method transformation: Parameterizes deformation using a weighted grid of control points of basis functions with local influence; the deformation is local, allowing translation in 3 N dimensionality. (6) Viscous/elastic/optical flow and finite element methods transformation: The physical transformation models, and the deformation is local, allowing translation in 3 N dimensionality. The DIR algorithm of this study was an intensity-based free-form DIR algorithm that was provided with the MIM software.

**Table 1:** Mathematical formulations of similarity metrics

	Category	Equation	Description
Geometry or Feature-based Metrics	Point matching	$\mathrm{R}=\sum {\left({p}_{A^{\prime }}-{p}_B\right)}^2/\mathrm{N}$	The registration metric R is defined as the sum of the squared distances between corresponding points ${p}_{A^{\prime }}\ \mathrm{and}\ {p}_B$, where N is the total number of points [16].
	Surface matching	$\mathrm{R}=\sum \mathrm{dist}{\left({p_{A^{\prime}}}^{'},{S}_B\right)}^2/\mathrm{N}$	The dist (${p}_{A^{\prime }}$, *S*_*B*_) computes the (minimum) distance between point ${p}_{A^{\prime }}$ and the surfaces *S*_*B*_, N is the number of points in Study A [16].
Intensity-based Metrics	Sum of Squared Differences (SSD)	$\mathrm{SSD}=\sum {\left({I}_{A^{\prime }}-{I}_B\right)}^2/\mathrm{N}$	The SSD metric is defined as the average squared intensity (${I}_{A^{\prime }}$ and *I*_*B*_) difference between Study A and Study B, where N is the number of evaluated voxels [16].
	Correlation Coefficient (CC)	CC=$\frac{\sum_{\overrightarrow{x}}\left(A\left(\overline{x}\right)-\overline{A}\right)\left(T\left(B\left(\overrightarrow{x}\right)\right)-\overline{B}\right)}{\sqrt{\sum_{\overrightarrow{x}}{\left(A\left(\overline{x}\right)-\overline{A}\right)}^2{\sum}_{\overrightarrow{x}}{\left(T\left(B\left(\overrightarrow{x}\right)\right)-\overline{B}\right)}^2}}$	CC measures the similarity in the image signal, which assumes a linear relationship between voxel intensities in two images [16].
	Mutual Information (MI)	$\mathrm{MI}\ \left({I}_{A^{\prime }},{I}_B\right)=\sum \limits_{\mathrm{B}}\sum \limits_Ap\left({I}_{A^{\prime }},{I}_B\right){\mathit{\log}}_2\left[p\left({I}_{A^{\prime }},{I}_B\right)/p\left({I}_{A^{\prime }}\right)p\left({I}_B\right)\right]$	MI has proven very effective for registering image data from different modalities, where *p* (I_A’_) and *p* (I_B_) are the probability distribution functions of the intensities I_A’_ and I_B_, respectively, and $p\left({I}_{A^{\prime }},{I}_B\right)$ is the joint probability distribution function [16].

To evaluate the effect of the DIR, the minimum and maximum volume phases (Volume _min_/Volume _max_) of the heart and the LVM, the minimum and maximum DSC phases (DSC _min_/DSC _max_) of the heart and LVM were selected in this study, in other words, the heart and LVM in the minimum and maximum volume phase, the heart and LVM in the minimum and maximum DSC value phase were respectively selected. First, the heart and LVM in the Volume _min_ phase were deformed to the Volume _max_ phase, obtaining the volumetric deformable heart and LVM (Volume _deformation_). Then, the relevant dose was deformed according to the deformation vector. Second, the heart and LVM in DSC _min_ phases (refer to the 0% phase) were deformed to the DSC _max_ phases, obtaining the morphological deformable heart and LVM (DSC _deformation_), and the relevant dose was deformed according to the deformation vector.

### Data analysis

In present study, manual calculation and automatic calculation with the above two methods were compared. For two extreme volumetric phases, the variation in the Volume _min_ and Volume _deformation_ relative to the Volume _max_ were compared for the heart and LVM, and the Volume _max_ was used as a reference. Then, the relevant difference in dose-volume parameters was analyzed. For two extreme morphological phases, the variation in DSC _min_ and DSC _deformation_ phases relative to the DSC _max_ phase were compared for the heart and LVM; the DSC _max_ phase was used as a reference phase, and then the relevant difference in dose-volume parameters was analyzed. The DSC of the heart and LVM in different phases in reference to the 0% phase were calculated by using the formula $\mathrm{DSC}=\frac{2\mid \mathrm{A}\cap \mathrm{B}\mid }{\mid \mathrm{A}\mid +\mid \mathrm{B}\mid }$, where A represents the volume in the 0% phase, and B represents the volume in the other phases (5–95%), which were used to describe the morphology of cardiac structures. The dose-volume parameters mainly included D_mean_, V_20_, V_30_ and V_40_.

### Statistical analyses

All data were analyzed using SPSS v19.0 software (SPSS Inc., Chicago, IL). All data were described by the mean ± standard deviation ($\overline{x}\pm s$). For comparisons of data between two groups, the Wilcoxon signed-rank test was used in this study. The differences were considered statistically significant when *p* < 0.05.

## Results

### The deformation results in different phases of the heart using the DIR technique

**Fig. 2:**
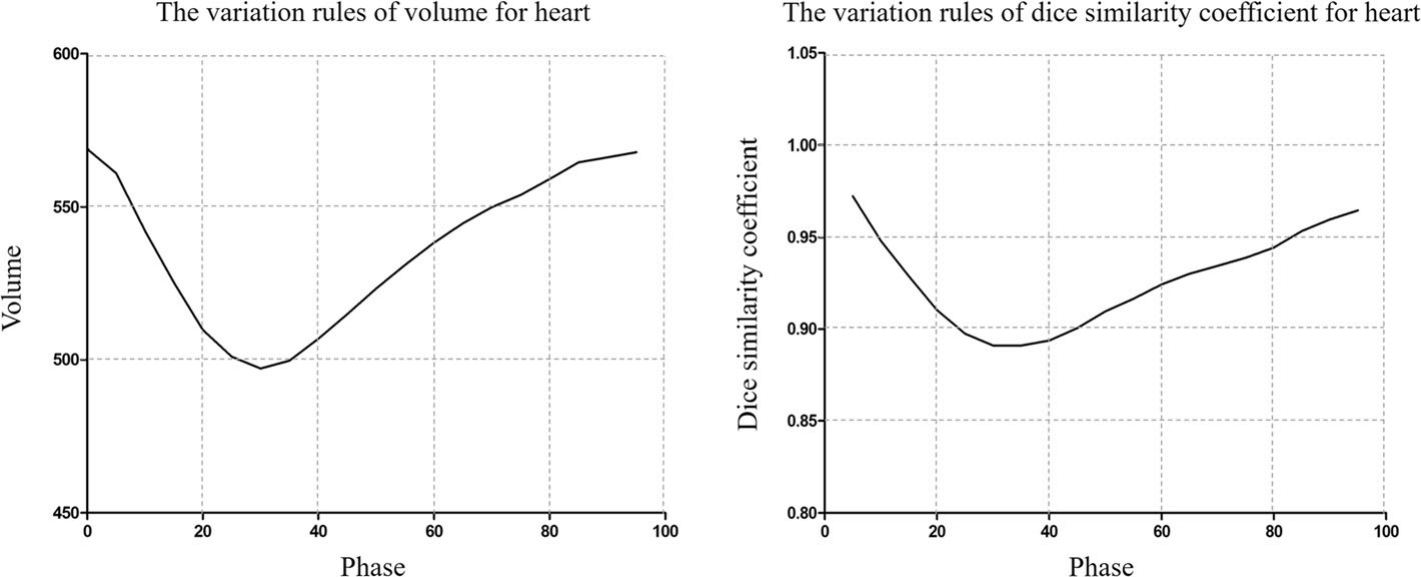
The variation in volume and dice similarity coefficient (DSC) for the heart

As shown in Fig. 2, the variation in volume and morphology of the heart presented good consistency in the cardiac cycle. The heart in the Volume _min_ phase was deformed to the Volume _max_ phase, and the difference between the pre- and post-registration Volume _min_ and Volume _max_ were reduced from (13.87 ± 2.84)% to (1.72 ± 1.45)% for the heart. There was statistical significance between the Volume _min_ and Volume _max_ (*p* < 0.05); however, there was no statistical significance between the Volume _deformation_ and Volume _max_ for the heart (*p >* 0.05). The dose-volume parameters such as D_mean_, V_20_, V_30_ and V_40_ of the heart were not significantly different between the Volume _min_ phase and Volume _max_ phase (*p* > 0.05), and these were significantly different between the Volume _deformation_ phase and the Volume _max_ phase (*p* < 0.05) (Table 2).

**Table 2:** Comparison of parameters in the Volume _min_, Volume _max_ and Volume _deformation_ phases for the heart

	Volume _min_	Volume _max_	Volume _deformation_	Volume _max_- Volume _min_	Volume _max_- Volume _deformation_	*P* (Volume _max_ vs Volume _min_)	*P* (Volume _max_ vs Volume _deformation_)
Volume (cm^3^)	496.63 ± 100.73	577.15 ± 119.28	576.24 ± 114.15	80.51 ± 25.32	9.99 ± 7.88	0.000	0.986
D_mean_	22.99 ± 2.70	22.89 ± 2.42	22.36 ± 2.46	0.41 ± 0.35	0.53 ± 0.34	0.487	0.000
V_20_	51.31 ± 6.89	51.55 ± 5.85	50.34 ± 6.08	1.30 ± 0.91	1.26 ± 0.77	0.476	0.000
V_30_	45.82 ± 6.71	46.01 ± 5.67	44.78 ± 5.91	1.26 ± 0.92	1.28 ± 0.78	0.498	0.000
V_40_	12.83 ± 6.21	12.72 ± 6.40	12.01 ± 6.03	0.75 ± 0.59	0.76 ± 0.70	0.476	0.001

The heart in DSC _min_ phases (refer to the 0% phase) were deformed to the DSC _max_ phases, the DSC in the DSC _deformation_ phase were increased from 0.899 ± 0.014 to 0.950 ± 0.009 compared to those in the DSC _min_ phase, which all refer to the DSC _max_ phase. In addition, the dose-volume parameters such as D_mean_, V_20_, V_30_ and V_40_ of the heart were not significantly different between the DSC _min_ phase and the DSC _max_ phase (*p* > 0.05); however, these were statistically significant between the DSC _deformation_ phase and the DSC _max_ phase (*p* < 0.05) (Table 3).

**Table 3:** Comparison of parameters in the DSC _min_, DSC _max_ and DSC _deformation_ phases for the heart

	DSC_min_	DSC_max_	DSC_deformation_	DSC_max_-DSC_min_	DSC_max_-DSC_deformation_	*P* (DSC _max_ vs DSC _min_)	*P* (DSC _max_ vs DSC _deformation_)
D_mean_	22.99 ± 2.70	22.76 ± 2.43	22.31 ± 2.49	0.45 ± 0.39	0.51 ± 0.31	0.079	0.001
V_20_	51.23 ± 6.84	51.26 ± 5.86	50.21 ± 6.20	1.26 ± 0.92	1.14 ± 0.68	0.986	0.000
V_30_	45.74 ± 6.66	45.71 ± 5.69	44.65 ± 6.04	1.22 ± 0.94	1.13 ± 0.71	0.958	0.000
V_40_	12.93 ± 6.24	12.51 ± 6.34	11.84 ± 6.07	0.81 ± 0.53	0.71 ± 0.56	0.050	0.000

### The deformation results in different phases of the LVM using the DIR technique

**Fig. 3:**
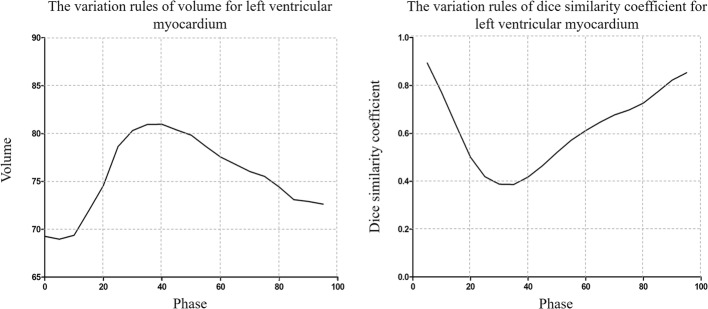
The variation in volume and dice similarity coefficient (DSC) for the left ventricular myocardium (LVM)

As shown in Fig. 3, the variations in volume and morphology of the LVM were not consistent in the cardiac cycle. The LVM in the Volume _min_ phase was deformed to the Volume _max_ phase; the difference between the pre- and post-registration Volume _min_ and Volume _max_ was reduced from (18.77 ± 6.64)% to (17.38 ± 7.89)% for the LVM; there were all significantly different between the Volume _min_/Volume _deformation_ and the Volume _max_ (*p* < 0.05). The dose-volume parameters such as D_mean_, V_20_, V_30_ and V_40_ of the LVM were statistically significant between the Volume _min_/Volume _deformation_ phase and the Volume _max_ phase (*p* < 0.05) (Table 4).

**Table 4:** Comparison of parameters in the Volume _min_, Volume _max_ and Volume _deformation_ phases for the left ventricular myocardium

	Volume _min_	Volume _max_	Volume _deformation_	Volume_max_-Volume_min_	Volume_max_- Volume _deformation_	*P* (Volume _max_ vs Volume _min_)	*P* (Volume _max_ vs Volume _deformation_)
Volume (cm^3^)	65.91 ± 14.91	81.69 ± 19.41	67.36 ± 17.01	15.78 ± 7.84	14.33 ± 7.47	0.000	0.000
D_mean_	9.88 ± 3.63	7.36 ± 4.28	8.35 ± 3.88	2.76 ± 1.64	1.33 ± 0.95	0.000	0.003
V_20_	19.08 ± 9.23	12.20 ± 11.17	14.62 ± 10.06	7.34 ± 4.80	3.36 ± 2.50	0.000	0.006
V_30_	14.35 ± 8.18	8.33 ± 9.49	10.49 ± 8.31	6.58 ± 4.69	3.14 ± 2.48	0.000	0.010
V_40_	2.89 ± 3.10	1.30 ± 2.81	2.07 ± 2.93	2.20 ± 2.06	1.35 ± 1.41	0.005	0.061

The LVM in the DSC _min_ phases (refer to 0% phase) was deformed to that in the DSC _max_ phases; the DSC in the DSC _deformation_ phase were increased from 0.389 ± 0.098 to 0.773 ± 0.052 compared to those in the DSC _min_ phase, which all refer to the DSC _max_ phase. In addition, the dose-volume parameters such as D_mean_, V_20_, V_30_ and V_40_ of the LVM were all significantly different between the DSC _min_/DSC _deformation_ phase and the DSC _max_ phase (*p* < 0.05) (Table 5).

**Table 5:** Comparison of parameters in the DSC _min_, DSC _max_ and DSC _deformation_ phases for the left ventricular myocardium

	DSC_min_	DSC_max_	DSC_deformation_	DSC_max_-DSC_min_	DSC_max_-DSC_deformation_	*P* (DSC _max_ vs DSC _min_)	*P* (DSC _max_ vs DSC _deformation_)
D_mean_	6.80 ± 4.06	11.12 ± 4.39	8.30 ± 3.68	4.45 ± 2.68	2.95 ± 2.53	0.000	0.000
V_20_	10.59 ± 10.11	21.24 ± 9.01	15.38 ± 9.43	10.99 ± 4.71	6.21 ± 3.15	0.000	0.000
V_30_	6.82 ± 8.27	16.27 ± 8.17	10.95 ± 8.01	9.86 ± 4.91	5.74 ± 3.23	0.000	0.000
V_40_	1.09 ± 2.82	3.51 ± 3.62	1.47 ± 2.70	2.75 ± 2.56	2.26 ± 2.40	0.000	0.001

## Discussion

This study analyzed the variation of dosimetry parameters for the heart and the LVM in different phases before and after deformation by applying the DIR algorithm from MIM software. In addition, recommendations were given when performing dose accumulation for the heart and LVM by applying the DIR technique.

The emergence of the DIR technique has laid the foundation for a reduction in contouring time and the realization of dose accumulation. Wang et al. found that the DIR algorithm could be an effective method to transfer regions of interest (ROIs) in the planned CT to subsequent CT images with altered anatomical structures [17]. Dam et al. indicated that the internal target volume (ITV), which was automatically contoured with the DIR, was more accurate and could reduce the difference between physicians in non-small cell lung cancer radiotherapy [13]. Based on these findings, we believe that the DIR technique has the potential to realize the actual dose calculation in the cardiac cycle. However, the underlying premise is that the effect of the DIR is sufficient; therefore, this study analyzed the deformation results of the DIR technique in the heart and LVM based on ECG-gated 4DCT data obtained in the breath-hold condition.

As commercial software for clinical applications, MIM has been proven to have a high-accuracy DIR algorithm, which based on an intensity-based free-form algorithm [18]. Because the two phases with the largest volume difference are not necessarily the ones with the largest morphological difference, we decided to perform the DIR in two groups. To evaluate the effect of the DIR in extreme volumetric and morphological variations, this study deformed the heart and LVM in the Volume _min_ /DSC _min_ phase to the Volume _max_ /DSC _max_ phase. Our results showed that in these two kinds of deformations, the volume and DSC of the heart were significantly improved after the DIR. However, the dose-volume parameters were all significantly different after deformation compared to those in the Volume _max_/DSC _max_ phase, which indicates that the dose-volume parameters after the deformation not only did not closer to those in the target phases but also had a wider gap with those in the target phases. This result indicated that there was no significant relationship between the cardiac dose variation and the cardiac volumetric and morphological variation, mainly because cardiac volumetric and morphological variation leads to location variation between the heart and the dose line. In addition, we found that there was a problem of excessive deformation in the apex cordis and at the top of the heart, which indicates that the local individualized DIR must be considered. All dose-volume parameters were not showed statistically significant differences before deformation, indicating that there was a smaller impact of cardiac activity on the cardiac dosimetric evaluation.

For the LVM, the volumetric variation was inconsistent with the morphological variation, and the volume and DSC of the LVM were insignificantly improved after the DIR. These results showed that compared with the LVM in the DSC _max_ phase, the DSC of the LVM in the DSC _deformation_ phase was 0.773, which according to the recently published recommendations of Task Group 132 is marginally outside the acceptable range [16]. Moreover, the dose-volume parameters of the LVM also showed less improvement when using the DIR of this study, which were all statistically significant before and after deformation compared to those in the Volume _max_/DSC _max_ phase. Thus, the DIR algorithm used in this study may not be ideal for the effect of the LVM, which might be related to the irregular geometry of the LVM. These findings indicate that, in terms of the DIR algorithm used in this study, as a result of the morphology of the LVM exhibiting irregularity and the significant morphological variation in the cardiac cycle, some difficulties may be encountered for accurate contouring, accurate calculation of the dose and the implementation of dose accumulation for the LVM. Although the deformation effect is related to the DIR algorithm, the result of this study still reminded us that the dose evaluation of the LVM need to be careful in applicating the DIR technology, and it is necessary for clinical practice to analyze the deformation precision of the LVM separately. In addition, the uncertainty of the evaluation in the LVM dose is more complex because of the inconsistency between the volumetric variation and the morphological variation; this inconsistency indicates that the DIR algorithm must be designed with multidimensional parameters, and the accuracy and efficiency are not high in the pure intensity-based DIR algorithm. Moreover, the variation of the LVM was more remarkable than in the heart, suggesting that individualized dose evaluations and limitations should be considered.

In this study, we innovatively analyzed the feasibility of applying the DIR technique to assess the dose of the heart and the LVM, our results lay a foundation for the realization of actual dose accumulation for the cardiac structure in the cardiac cycle. In the future, our research group will further study parameters that could be added to the DIR algorithm to improve the accuracy of dose deformable registration, in addition, the clinical application of the DIR technique in improving the accuracy of the cardiac dose calculation during the cardiac cycle compared with traditional static three-dimensional CT (3DCT) will also be considered in the next step.

## Conclusions

There was no significant relationship between the variation in dose-volume parameters and the variation in the volume and morphology for the heart, moreover, because of the presence of excessive deformation, the local individualized registration should be considered in cardiac DIR. However, the inconsistency of the volumetric variation and the morphological variation for the LVM may be major factors leading to uncertainty in dose-volume evaluation, this inconsistency indicates that the local DIR algorithm with multidimensional parameters must be designed.

## Abbreviations

3DCT: Three-dimensional computed tomography
4DCT: Four-dimensional computed tomography
DIR: Deformable image registration
DSC: Dice similarity coefficient
ECG: Electrocardiograph
ITV: Internal target volume
LVM: Left ventricular myocardium
OAR: Organ at risk
RIHD: Radiation-induced heart disease
ROI: Regions of interest
